# Pectate lyase genes from *Radopholus similis* and their application in pathotype identification

**DOI:** 10.1007/s00253-024-13124-3

**Published:** 2024-04-12

**Authors:** Sihua Yang, Shuai Yang, Qianying Li, Yang Lu, Xin Huang, Chun Chen, Chunling Xu, Hui Xie

**Affiliations:** Laboratory of Plant Nematology and Research Center of Nematodes of Plant Quarantine, Department of Plant Pathology, College of Plant Protection, South China Agricultural University, Guangzhou, 510642 People’s Republic of China

**Keywords:** *Radopholus similis*, Pathotypes, Molecular identification, Pectate lyase, Tropism

## Abstract

**Abstract:**

*Radopholus similis* is a destructive, migratory, and endophytoparasitic nematode. It has two morphologically indistinguishable pathotypes (or physiological races): banana and citrus pathotypes. At present, the only reliable method to differentiate the two pathotypes is testing the infestation and parasitism of nematodes on *Citrus* spp. via inoculation. However, differences in inoculation methods and conditions adopted by different researchers complicate obtaining consistent results. In this study, the parasitism and pathogenicity of 10 *R. similis* populations on rough lemon (*Citrus limon*) seedlings and the tropism and invasion of rough lemon roots were tested. It revealed that populations SWK, GJ, FZ, GZ, DBSR, and YJ were citrus pathotypes, which showed parasitism and pathogenicity on rough lemon and could invade rough lemon roots, whereas populations XIN, ML, HN6, and HL were banana pathotypes, having no parasitism and pathogenicity on rough lemon and they did not invade the rough lemon roots. Four pectate lyase genes (*Rs-pel-2, Rs-pel-3, Rs-pel-4*, and *Rs-pel-5*) belonging to the Class III family from these populations were amplified and analysed. The gene *Rs-pel-3* could be amplified from six citrus pathotype populations and was stably expressed in the four developmental stages of the nematode, whereas it could not be amplified from the four banana pathotypes. *Rs-pel-3* expression may be related to the parasitism and pathogenicity of *R. similis* on rough lemon. Hence, it can be used as a molecular marker to distinguish between banana and citrus pathotypes and as a target gene for the molecular identification of these two pathotypes.

**Key points:**

*• Four pectate lyase genes (Rs-pels) from Radopholus similis were cloned and analysed.*

*• The expression of Rs-pels is different in two pathotypes of Radopholus similis.*

*• A molecular identification method for two pathotypes of Radopholus similis using pectate lyase gene Rs-pel-3 as the target gene was established.*

**Supplementary Information:**

The online version contains supplementary material available at 10.1007/s00253-024-13124-3.

## Introduction

*Radopholus similis*, with a common name, the burrowing nematode, a migratory endophytoparasitic nematode, is one of the top 10 most damaging plant-parasitic nematodes worldwide (Jones et al. 2013). *R. similis* has over 350 host plants, including bananas, *Citrus* spp., and ornamental plants, and is an important factor in reducing the production of bananas and other horticultural crops worldwide; it is listed as a quarantine plant pest in most countries (Xie 2006; Duncan and Mons 2013). Two pathotypes (or physiological races) of *R. similis* are generally recognised: the banana and citrus pathotypes. The banana pathotype parasitises bananas and many other hosts, but not *Citrus* spp., whereas the citrus pathotype parasitises both bananas and *Citrus* spp. (Valette et al. 1998). Therefore, the citrus pathotype has a wider host range and is more harmful than the banana pathotype. The European and Mediterranean plant Protection Organizations and European Union classify banana and citrus pathotypes as A1 and A2 quarantine pests, respectively (EPPO 2008). At present, the mechanisms of host specificity and differences in pathogenicity between these two pathotypes are unclear. Testing the parasitism of nematodes on *Citrus* spp. is recognised as the only reliable method for distinguishing between these pathotypes.

Pectate lyase (PEL) degrades pectin to produce oligosaccharides via β-trans elimination and exists widely in plants and microorganisms (Chen et al. 2019). Pathogenic microorganisms destroy plant cell walls to facilitate their invasion via secreting PEL to degrade pectin and stimulate plant immune responses (Shevchik et al. 1998; Ferrari et al. 2013). Therefore, PEL plays an important role in the interaction between plant pathogens and their hosts and is an important factor affecting the host range of pathogens (Filho et al. 2016). Currently, plant-parasitic nematodes are the only animals known to produce PEL. The first PEL gene (*pel*) found in plant-parasitic nematodes was *Gr-pel-1* from *Globodera rostochiensis* (Popeijus et al. 2000). Subsequently, several *pel* genes were identified and cloned from sedentary parasitic nematodes belonging to the genera *Heterodera*, *Globodera*, and *Meloidogyne*, all of which belong to the Class III PEL family (Deboer et al. 2002; Doyle et al. 2002; Huang et al. 2005; Kudla et al. 2007; Vanholme et al. 2007; Zhuo et al. 2011; Peng et al. 2012, 2016; Li et al. 2017). The PEL of sedentary parasitic plant-parasitic nematodes may play an important role in host specialization and pathogenic differentiation (Huang et al. 2005; Stare et al. 2011; Sabeh et al. 2019; Tian et al. 2019). To date, only three *pel* genes have been identified and cloned from *Bursaphelenchus xylophilus*, a migratory parasitic nematode (Kikuchi et al. 2006; Lee et al. 2013); however, the function and mechanism of these genes and their relationship with pathogenic differentiation have not been reported.

In this study, five *pel*s from *R. similis* (*Rs-pel*s) were screened and cloned using transcriptome data (Huang et al. 2019), and their basic characteristics were studied using bioinformatics. The pathogenicity and tropism of 10 *R. similis* populations from different hosts were tested to determine their pathotypes against rough lemon (*Citrus limon*). Then, the expression of *Rs-pel*s in these populations was analysed to screen the differential *pel* genes that could potentially distinguish between the banana and citrus pathotypes to establish a molecular identification method for these two pathotypes.

## Materials and methods

Ten populations of *R. similis* and their host sources are shown in Table 1. All populations were identified by the Plant Nematodes Laboratory of South China Agricultural University, propagated and preserved on carrot callus (25°C) according to the method described by Fallas and Sarah (1994). *R. similis* was isolated from carrot callus by the method of Stoffelen et al. (1999), soaked in 0.2% streptomycin sulfate for 8 hours, and then washed with sterile distilled water for five times, and then the nematode suspensions were obtained for subsequent experiments. The different developmental stages (eggs, juveniles, females, and males) of *R. similis* were distinguished by morphological characteristics under a Nikon SMZ18 stereomicroscope (Nikon Corporation, Tokyo, Japan). Females and males have vulva and spicule respectively.

**Table 1:** Population information of *Radopholus similis*

No.	Population coding	Hosts
1	XIN	*Zingiber officinale* Roscoe
2	ML	*Crataegus pinnatifida*
3	SWK	*Chrysalidocarpus lutescens*
4	HN6	*Musa* AAA Giant Cavendish cv. Baxi
5	HL	*Maranta arundinacea*
6	GJ	*Citrus reticulata*
7	FZ	*Anthurium andraeanum* ‘Pink Champion’
8	GZ	*Anthurium andraeanum Linden*
9	YJ	*Curcuma longa*
10	DBSR	*Anubias nana*

The *C. limon* seeds were donated by Prof. Xiaoling Deng from South China Agricultural University. River sand purchased from Guangzhou Xiankelian Garden Flower Co., Ltd. (Guangzhou, China) was used as the planting medium. A glass tube (3 cm × 15 cm) was filled with dry river sand, accounting for about 1/3 the tube height, and was sterilised at 125 °C 20 p.s.i (about 137.8 kPa) for 1 h; after cooling overnight, it was subjected to secondary sterilisation under the same conditions. After peeling the seed coat, rough lemon seeds were placed in 0.26% sodium hypochlorite solution for 30 min, washed with sterile water four times, laid flat in a Petri dish (d=90 mm) with moist sterilised filter paper at the bottom, and placed in an incubator at 25 ± 1.5 °C for 3–4 days to accelerate germination in the dark.

Sterile distilled water (3 mL) was added to each sand-filled tube to moisten the sand and a 2 cm deep depression was placed in the sand surface centre; a single seed was placed in it, and covered with sterile sand. The tubes were then placed in an incubator at 25 ± 1.5 °C with the light intensity of 3200–4000 l × (9.5 h light and 14.5 h darkness), normal watering management, and humidity maintained at approximately 3% w/w. After 30 days of culture, the rough lemon seedlings were inoculated.

### Determining parasitism and pathogenicity of tested nematodes to rough lemon

As per the method described by Kaplan (1994), rough lemon seedlings with essentially the same growth after planting for 30 days were selected and inoculated nematode suspensions by a dropper with an inoculation amount of 200 female nematodes (identified under a Nikon SMZ18 stereomicroscope; Nikon Corporation, Tokyo, Japan) per plant. Inoculation was performed twice at an interval of 5 days, and 100 nematodes were inoculated each time. Each population of *R. similis* was inoculated for one treatment, and each treatment was repeated five times; non-inoculated seedlings were used as a control treatment. Inoculation tests were repeated twice. The day before inoculation, sterile distilled water was added to each test tube to wet the sand, and the nematode suspension was inoculated at a depth of 1 cm underground. To ensure normal infection of nematodes, plants were not watered for the first three days after inoculation, and routine management was performed. After inoculation with nematodes for 30 days, the inoculated plant root systems were observed and photographed, plant symptoms were recorded, plant height and root weight were measured, and the nematodes were isolated according to Kaplan’s method and counted using a Nikon SMZ18 stereomicroscope (Nikon Corporation, Tokyo, Japan).

### Tropism and invasion of nematodes to the rough lemon root system

The root systems of rough lemon seedlings were cut into 1 cm long segments. According to Čepulytė et al. (2018), Pluronic F-127 gel system was prepared to conduct the tropism test of *R. similis* to the rough lemon root. This gel was liquid at low temperatures (< 15 °C) but solidified into a gel at higher temperatures (> 15 °C). At the temperature of 10 °C, 1 mL of Pluronic F-127 gel was absorbed into a sterile 24-well culture plate. A total of 200 female nematodes were picked up from nematode suspensions by a needle under a Nikon SMZ18 stereomicroscope and added to each well, gently shaken and mixed, and then a fresh rough lemon root segment was placed in each well. The culture plate was sealed and transferred to a dark incubator at 28 °C for culturing. Each nematode population had five wells (five replicates), and the experiment was repeated twice. Nematode tropism was observed under a Nikon SMZ18 stereomicroscope after 2, 4, 6, and 8 h of culturing. The nematode aggregation and invasions around the root incision were photographed and recorded. In addition, a modified sodium chlorate-acid fuchsin staining method (Feng 2001) was used to dye the root segments cultured for 8 h. Stained root tissues were sliced and photographed under a Nikon SMZ18 stereomicroscope.

### Cloning of *pel* genes from *R. similis*

A total of 20,000 mixed-stage nematodes of the GJ population were isolated and collected from a carrot callus in which *R. similis* was cultured. The total RNA of the nematodes was extracted by a Trizol Regent (Invitrogen, Carlsbad, California, USA). The 5' RACE and the 3' RACE cDNA templates for RACE amplification were synthesized by the SMART RACE cDNA amplification kit (Clontech, Takara Biotechnology (Dalian) Co., Ltd., Dalian, China), respectively. The cDNA template for the open reading frames (ORF) sequence amplification was synthesized by One-step gDNA Removal and cDNA synthesis SuperMix Kit (Transgen, Beijing, China). The total genomic DNA of *R. similis* was extracted by HiPure Mollusc DNA Kit (Magen Co., Ltd., Guangzhou, China).

Screening and blasting of the transcriptome data of *R. similis* (Huang et al. 2019) were performed to obtain the suspected *pel*s sequences. Primers for RACE amplification of *pel*s were designed according to those sequences (Supplementary Information). The 5' and the 3' terminal fragments of *pel*s were amplified using these primers, with the 5' RACE cDNA and the 3' RACE cDNA serving as templates for amplification, respectively. The amplified fragments were sequenced and spliced together by Sangon Biotech Co., ltd (Shanghai, China). The ORFs of *Rs-pel*s (*Rs-pel-1, Rs-pel-2, Rs-pel-3, Rs-pel-4, Rs-pel-5*) were predicted on an ORF finder website (http://www.ncbi.nlm.nih.gov/gorf/orfig.cgi), and a pair of full-length primers were designed for each *Rs-pel* (Supplemental Material, Table S1) by Primer Premier 6 software (PREMIER Biosoft International, Palo Alto, CA, USA). The ORF and genomic DNA sequences of *Rs-pel*s were amplified using full-length primers, with the cDNA and genomic DNA serving as templates for amplification, respectively.

The similarity of *Rs*-PELs was compared to the NCBI database using the BlastX tool (http://blast.ncbi.nlm.nih.gov/Blast.cgi). Signalp-4.0 (http://www.cbs.dtu.dk/services/signalp-4.0/) and TMHMMServer v.2.0 (https://services.healthtech.dtu.dk/services/TMHMM-2.0/) were used to predict the signal peptide (SP) and transmembrane domains of *Rs*-PELs, respectively. Multiple sequence alignments of *Rs*-PEL and other PELs from other nematodes were performed using Mega 6.0 (http://www.megasoftware.net/), and a phylogenetic tree was constructed using the maximum-likelihood method.

### Expression determination of *Rs-pel*s in *R. similis* populations

Using *Rs-pel*s as the target, the DNA of a single nematode from each population was extracted for polymerase chain reaction (PCR) detection, and the expression of *Rs-pel*s in different populations was analysed and determined. The target genes were selected for identification. To analyse the stable expression of the target gene, DNA from four developmental stages (eggs, juveniles, females, and males) was used as a template for PCR amplification to establish a method for rapid identification of the pathotypes of *R. similis*. The PCR amplification of single nematode DNA was performed as described by Xu et al. (2016).

### Data processing and analysis

The SPSS 14.0 Software (SPSS Inc., Chicago, IL, USA) and Excel 2013 Software (Microsoft Inc., Washington, D.C.) were used for statistical analyses. One-way analysis of variance (ANOVA) was employed for the analysis of the mean and standard error, and Duncan’s Multiple Ranger Test (DMRT) was used for multiple comparisons. The significance of the difference was analysed at the level of *p*=0.05. Preliminary analyses showed that there was no significant difference (*p*>0.05) between the data from two runs of each experiment using dependent-sample *t* tests and that allowed data from two runs to be combined for analyses.

## Results

### Parasitism and pathogenicity of 10 *R. similis* populations to rough lemon

**Fig. 1:**
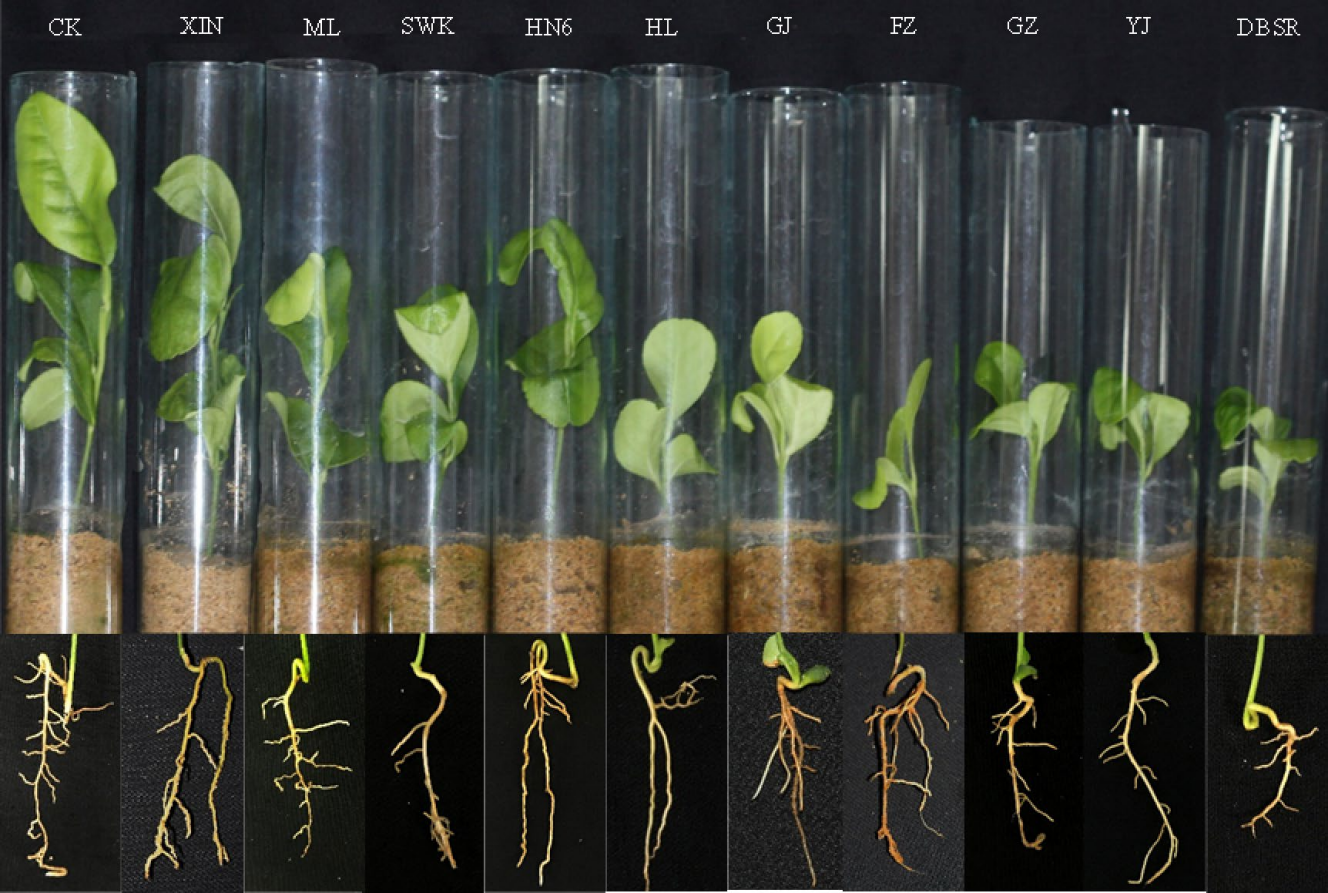
Symptoms of *Citrus limon* infected with 200 female nematodes of *Radopholus similis* per plant for 30 days. CK: non-inoculated rough lemon seedling and root system; XIN, ML, SWK, HN6, HL, GJ, FZ, GZ, YJ and DBSR: the rough lemon seedlings and root systems were inoculated with the different populations of *R. similis*, which originated from *Zingiber officinale* Roscoe (XIN), *Crataegus pinnatifida* (ML), *Chrysalidocarpus lutescens* (SWK), *Musa* AAA Giant Cavendish cv.Baxi (HN6), *Maranta arundinacea* (HL), *Citrus reticulata* (GJ), *Anthurium andraeanum* ‘Pink Champion’ (FZ), *Anthurium andraeanum* Linden (GZ), *Curcuma longa* (YJ), and *Anubias nana* (DBSR), respectively

Thirty days after inoculation, the root systems of rough lemons inoculated with the SWK, GJ, FZ, GZ, YJ, and DBSR populations were weak and had evident brown spots (Fig. 1). Plant height, root length, and root weight of the six treatments were significantly lower than those of the control (*p*<0.05) and the number of nematodes in the root and rhizosphere soils was significantly higher than those in the control (*p*<0.05). However, the average nematode number in the roots of the XIN, ML, HN6, and HL inoculation treatments was only 1–2, and the nematode number in the rhizosphere soil was less than 10, which was not significantly different from that of the control (*p*>0.05). The root length difference between these four treatments was also not significant, and the root length and weight of XIN and HN6 inoculation treatments were not significantly different from those of the control (*p*>0.05) (Table 2). Therefore, SWK, GJ, FZ, GZ, YJ, and DBSR populations could parasitise rough lemons and had clear pathogenicity, whereas XIN, ML, HN6, and HL populations did not parasitise rough lemons.

**Table 2:** Effects of *Radopholus similis* on *Citrus limon* growth 30 days after inoculation with 200 nematodes and the population density of the nematode recovered from root

Population	Height	Root length	Root weight	Number of nematodes in rhizosphere soil	Number of nematodes in root	Total number of nematodes
CK	8.86±0.36 f	7.52±0.32 f	0.11±0.01 d	0.00±0.00 a	0.00±0.00 a	0.00±0.00 a
XIN	7.72±0.39 e	6.75±0.38 ef	0.10±0.01 d	6.20±0.49 abc	1.80±0.47 a	8.00±0.58 ab
ML	6.11±0.42 cd	6.26±0.36 de	0.07±0.01 c	3.50±0.45 ab	1.40±0.50 a	4.90±0.67 ab
SWK	6.66±0.37 d	4.85±0.33 ab	0.05±0.01 abc	10.80±0.88 bc	9.50±0.75 b	20.30±0.80cd
HN6	6.86±0.26 de	6.16±0.40 de	0.10±0.01 d	6.10±1.39 abc	2.10±0.55 a	8.20±1.65 ab
HL	5.29±0.26 bc	6.44±0.60 de	0.07±0.00 c	8.30±0.47 abc	2.10±0.60 a	10.40±0.86bc
GJ	4.91±0.28 ab	5.03±0.32 abc	0.04±0.00 ab	10.80±0.63 bc	9.70±0.98 b	20.50±1.46cd
FZ	4.82±0.28 ab	6.02±0.30 cde	0.06±0.01 bc	33.10±5.37 e	20.40±1.99 c	53.50±5.84 e
GZ	4.91±0.26 ab	5.84±0.18 bcde	0.05±0.01 abc	20.00±1.97 d	9.90±1.11 b	29.90±2.69 d
YJ	4.51±0.23 ab	5.58±0.18 bcd	0.05±0.01 abc	13.60±1.55 cd	7.90±0.96 b	21.50±1.54 d
DBSR	4.26±0.30 a	4.27±0.14 a	0.04±0.00 a	12.50±0.91 cd	8.30±1.09 b	20.80±1.21 d

*CK*, healthy control group; *XIN, ML, SWK, HN6, HL, GJ, FZ, GZ, YJ and DBSR*, the treatments inoculated with the populations of *R. similis* originated from *Zingiber officinale* Roscoe (XIN), *Crataegus pinnatifida* (ML), *Chrysalidocarpus lutescens* (SWK), *Musa* AAA Giant Cavendish cv.Baxi (HN6), *Maranta arundinacea* (HL), *Citrus reticulata* (GJ), *Anthurium andraeanum* ‘Pink Champion’ (FZ), *Anthurium andraeanum* Linden (GZ), *Curcuma longa* (YJ), and *Anubias nana* (DBSR), respectively

### Tropism and invasion of 10 *R. similis* populations to the root system of rough lemon

**Fig. 2:**
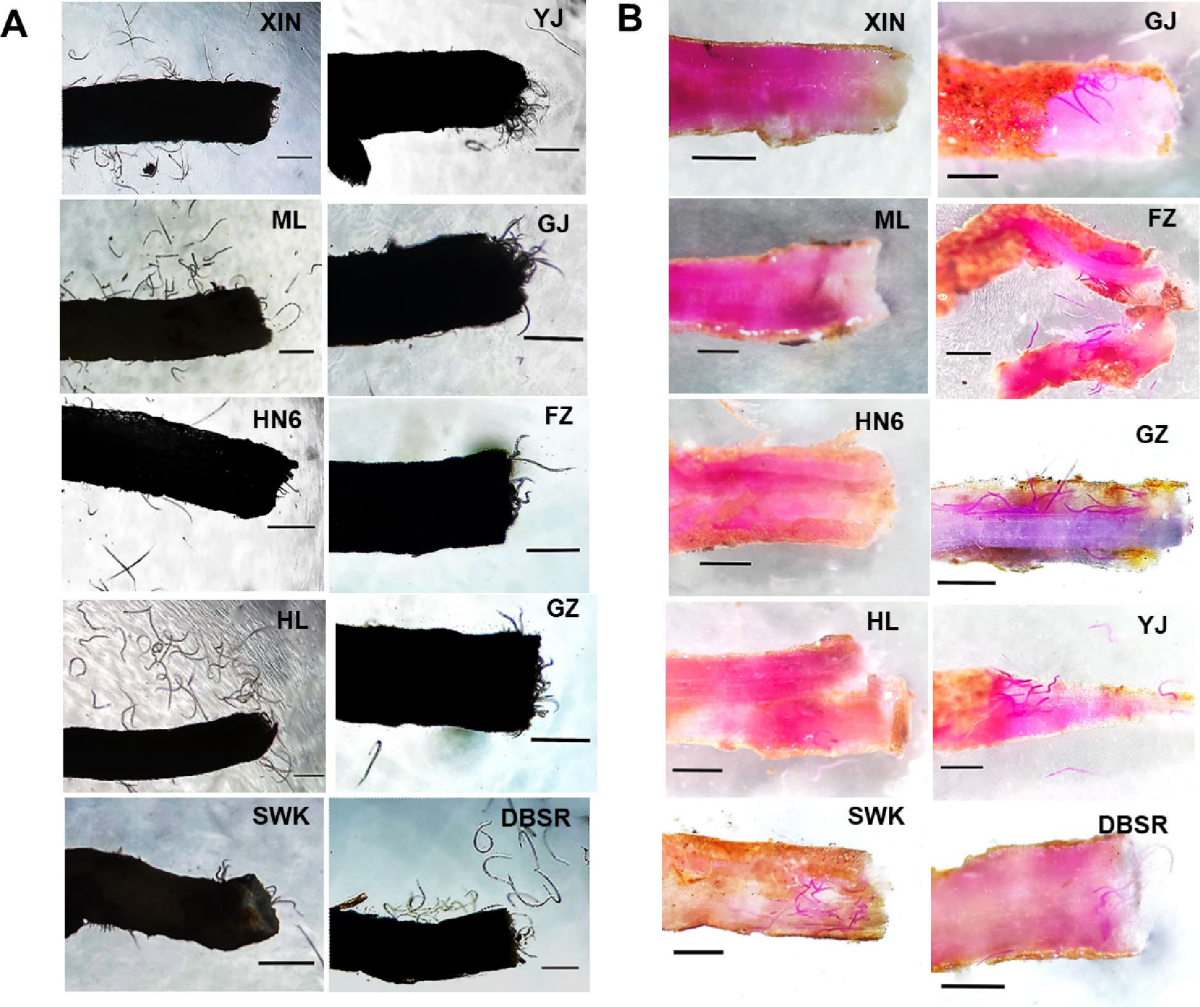
Tropism of 10 populations of *Radopholus similis* to the *Citrus limon* root. A: Tropism of *R. similis* to the root of *C. limon* in Pluronic F-127 gel evenly mixed with 200 *female nematodes* 6 h after assay initiation; B: Fuchsin staining of *C. limon* roots 8 h after assay initiation *by infection with 200 female nematodes*; XIN, ML, SWK, HN6, HL, GJ, FZ, GZ, YJ, and DBSR: the *C. limon* roots were infected with populations of *R. similis,* which originated from *Zingiber officinale* Roscoe (XIN), *Crataegus pinnatifida* (ML), *Musa* AAA Giant Cavendish cv.Baxi (HN6), *Maranta arundinacea* (HL), *Chrysalidocarpus lutescens* (SWK), *Citrus reticulata* (GJ), *Anthurium andraeanum* ‘Pink Champion’ (FZ), *Anthurium andraeanum* Linden (GZ), *Curcuma longa* (YJ), and *Anubias nana* (DBSR), respectively; scale bar = 500 μm

The tropism test of 10 *R. similis* populations to the rough lemon root system showed that the nematodes of all populations were randomly and uniformly dispersed in the gel when the root segment was placed in the well, and the nematodes started to migrate and aggregate to the root segment after 2 h and 4 h. The nematodes of the SWK, GJ, FZ, GZ, DBSR, and YJ populations were observed to invade the root from the wound position after 6 h, whereas the nematodes of the XIN, ML, HN6, and HL populations were still around the root and did not invade it (Fig. 2A). The root tissues treated for 8 h were dyed with modified sodium chlorate-acid fuchsin. Under the stereomicroscope, many nematodes of SWK, GJ, FZ, GZ, DBSR, and YJ populations were observed in the root segments, but only one or no nematodes of the XIN, ML, HN6, and HL populations were found in the root segments (Fig. 2B). The results showed that all the tested populations tended towards the rough lemon root; however, the SWK, GJ, FZ, GZ, DBSR, and YJ populations could invade the rough lemon root, whereas the XIN, ML, HN6, and HL populations did not invade the rough lemon root.

### Cloning and analysis of genes of the *pel* family in *R. similis*

**Fig. 3:**
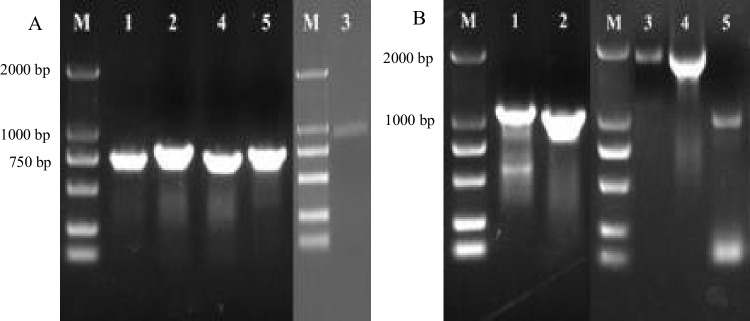
PCR amplification of five pectate lyase genes from *Radopholus similis* (*Rs-pel*s). **A**: Amplification of the ORF sequences of *Rs-pel*s; **B**: Amplification of the genomic DNA sequences of *Rs-pel*s. M:DS2000 marker (GDSBio Co., Ltd., Guangzhou China); 1–5: products of *Rs-pel-1*, *Rs-pel-2*, *Rs-pel-3*, *Rs-pel-4, Rs-pel-5* amplification respectively

Five transcripts of suspected *pel*s were obtained via screening and blasting the transcriptome data of the GJ population of *R. similis.* The ORF and DNA genomic sequences of these genes were obtained using PCR amplification (Fig. 3).

**Fig. 4:**
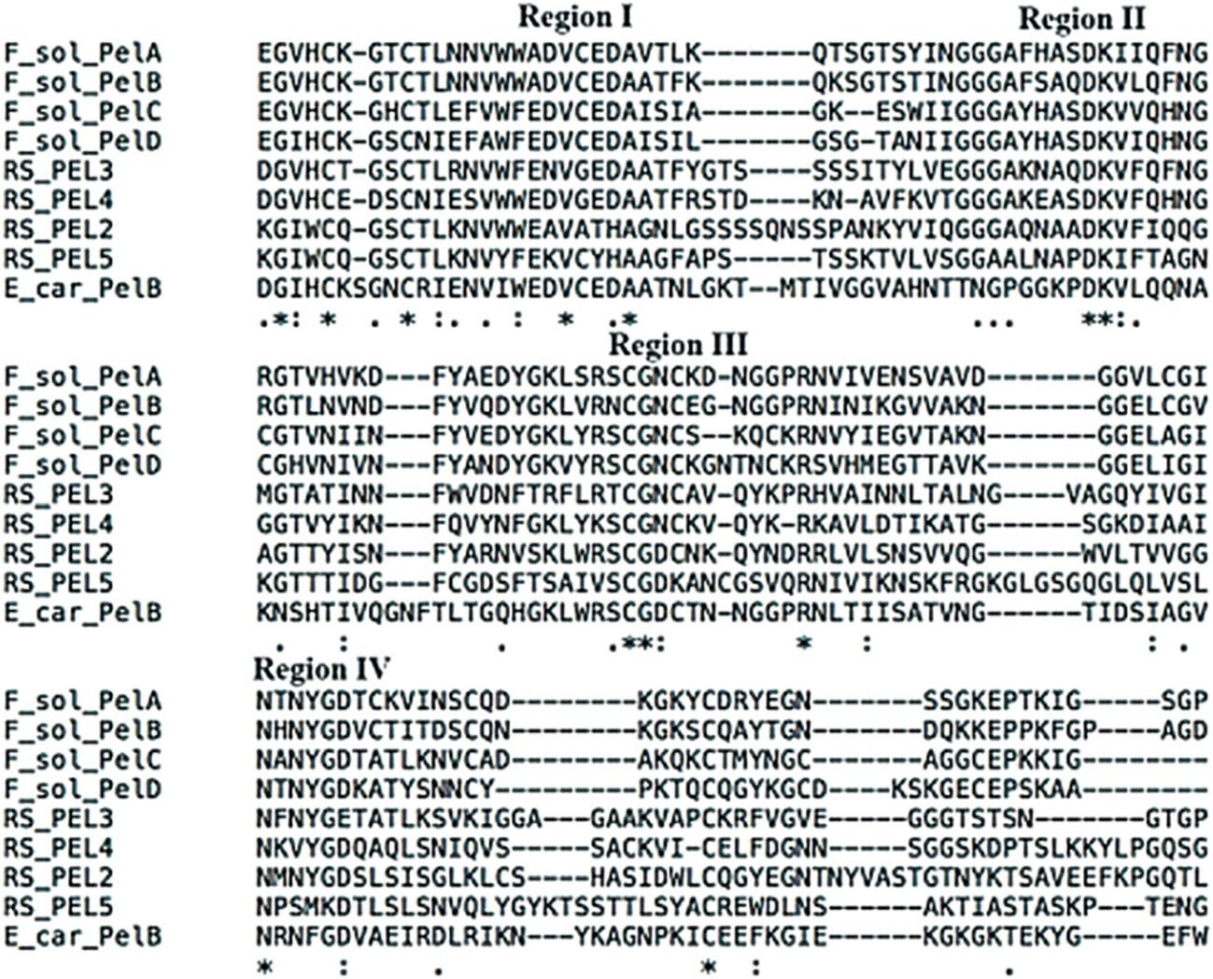
Predicting the conserved regions of pectate lyases of the Class III PEL family from *Radophulus similis*. Regions I–IV indicate conserved regions characteristic of the Class III pectate lyase family (Shevchik et al.1997). Highly conserved charged residues are indicated with asterisks (*), RS_PEL2 to 5 indicate the amino acid sequences of *Rs*-PEL-2 to *Rs*-PEL-5 from *R. similis,* the species of the bacterium or the fungus, the gene, the amino acid size, and the accession number of the aligned sequences are: F_sol_PelA = *Fusarium solani* f. sp. *pisi*, PelA, 242 aa (M94692.1); F_sol_PelB = *F. solani* f. sp. *pisi*, PelB, 242 aa (U13051); F_sol_PelC = *F. solani* f. sp. *pisi*, PelC, 215 aa (U13049); F_sol_PelD = *F. solani* f. sp. *pisi*, PelD, 233 aa (U13050); E_car_PelB = *Erwinia carotovora*, PelB, 347 aa (X79232)

After sequencing these fragments, a BLAST comparison confirmed that the deduced proteins of all five genes had conserved domains of the PEL family, and these five *pel*s were named *Rs-pel-1*, *Rs-pel-2*, *Rs-pel-3*, *Rs-pel-4*, and *Rs-pel-5*. Their ORFs were 843, 819, 873, 771, and 810 bp long, and the number of encoded amino acids (aa) were 280, 272, 290, 256, and 269 aa, respectively (Table 3). ClustalX was used to compare the amino acid sequences of these five *Rs*-PELs with known Class III PEL sequences, and *Rs*-PEL-2, *Rs*-PEL-3, *Rs*-PEL-4, and *Rs*-PEL-5 were confirmed to have four conserved regions unique to the Class III PEL family (Fig. 4) (Shevchik et al. 1997), whereas *Rs*-PEL-1 did not have a conserved region unique to Class III PEL.

**Table 3:** Sequence analyses of pectate lyases of *Radopholus similis*

Gene name	ORF length (bp)	Amino acid length (aa)	Protein molecular weight(kDa)	Signal peptide	Transmembrane domain	Genbank
*Rs-pel-1*	843	280	30.2	No	No	MN176119
*Rs-pel-2*	819	272	28.8	Yes	No	MN176120
*Rs-pel-3*	873	290	30.6	No	No	MN176121
*Rs-pel-4*	771	256	26.9	Yes	No	MN176122
*Rs-pel-5*	810	269	27.8	Yes	No	MN176123

Sequence analysis of the five *Rs*-PELs predicted that none of them had a transmembrane domain. Among them, *Rs*-PEL-2, *Rs*-PEL-4, and *Rs*-PEL-5 contained signal peptides, whereas *Rs*-PEL-1 and *Rs*-PEL-3 did not have signal peptide sequences (Table 3).

**Fig. 5:**
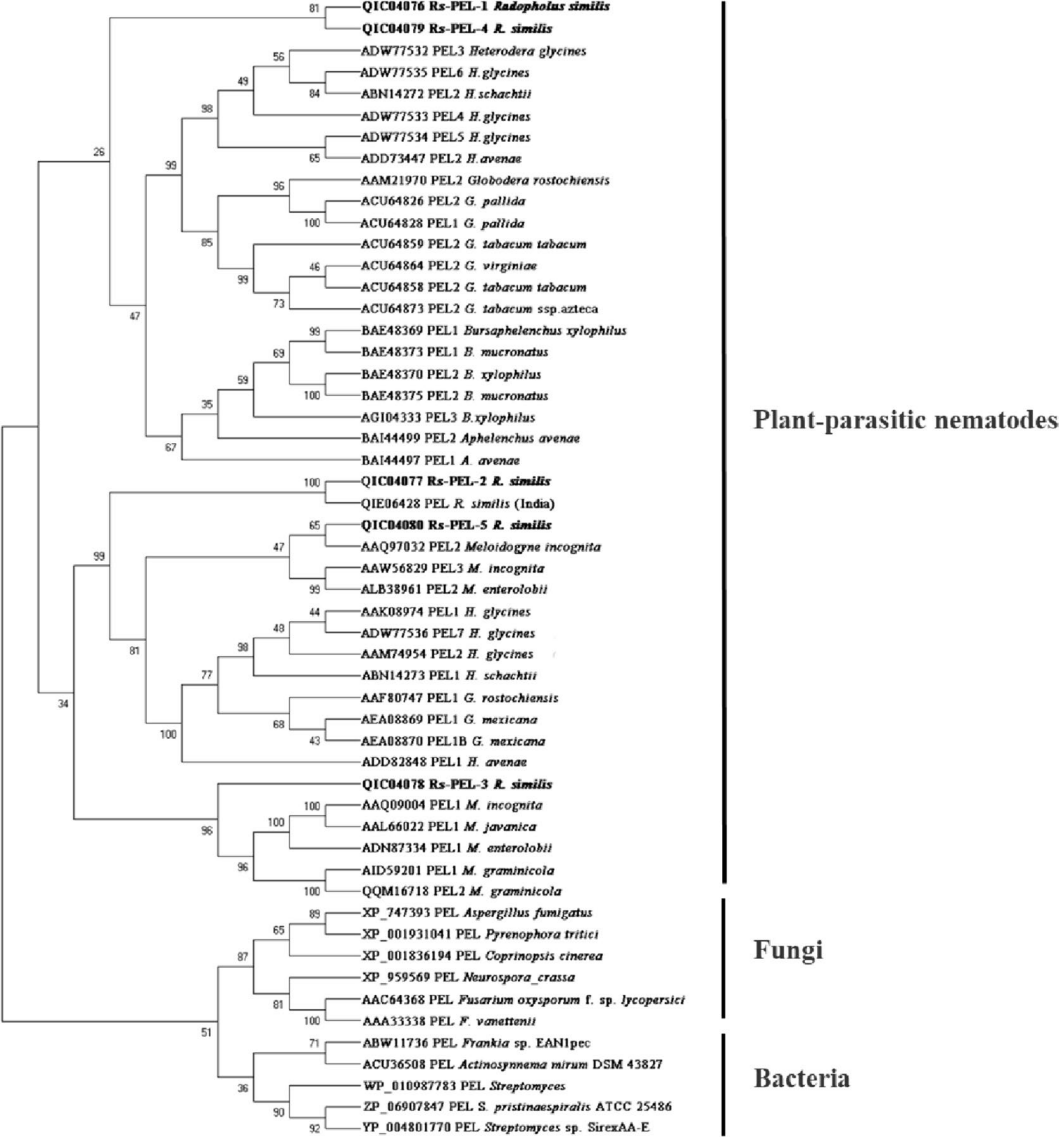
Maximum-likelihood phylogenetic trees of pectate lyases of *Radopholus similis* (*Rs-*PELs) and other organisms. Phylogenetic tree for proteins with conserved domains of pectate lyases from cyst nematodes, root-knot nematodes, *Aphelenchus*, *Bursaphelenchus*, bacteria, and fungi generated by MEGA6.0. The *Rs*-PELs amino acid sequences were marked in bold, and each sequence was followed by its accession number in GenBank

Amino acid sequences of the five *Rs*-PELs were blasted against those of other plant-parasitic nematodes, bacteria, and fungi, and a phylogenetic tree was constructed using the maximum likelihood method in MEGA software (Fig. 5). The results showed that PELs from fungi and bacteria clustered into a large category, whereas those from plant-parasitic nematodes clustered independently into another large category. The *Rs*-PELs could be divided into three categories: first, *Rs*-PEL-3 and the PELs from four species of *Meloidogyne* were clustered into a branch; second, *Rs*-PEL-2 and *Rs*-PEL-5 were clustered with the PELs from two species of *Meloidogyne* and species of *Heterodera* and *Globodera* into a branch; third, *Rs*-PEL-1 and *Rs*-PEL-4 were clustered with the PELs from the nematodes of *Heterodera*, *Globodera*, and Aphelenchida into one branch.

### Expression of PEL genes in 10 populations of *R. similis*

**Fig. 6:**
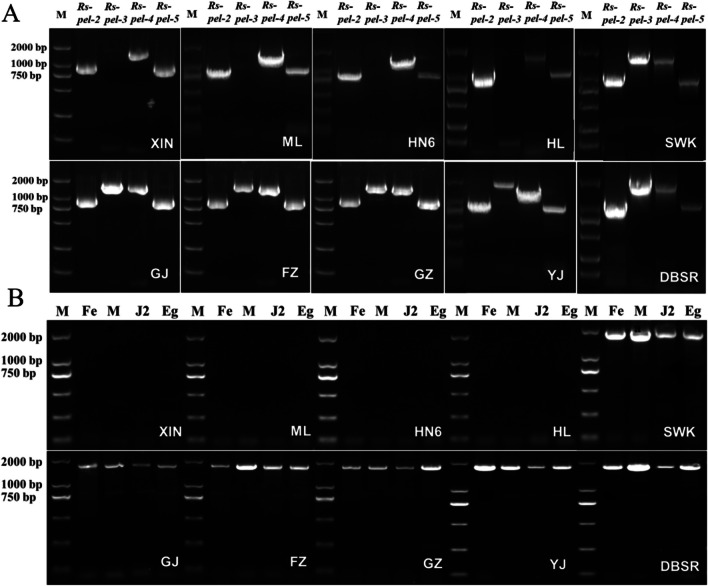
DNA amplification of pectate lyase genes of *Radopholus similis* (*Rs-pel*s) in 10 populations. A: DNA amplification of *Rs-pel*s in 10 populations of *R. similis*; B: DNA amplification of *Rs-pel-3* in different populations and developmental stages of *R. similis*; XIN, ML, SWK, HN6, HL, GJ, FZ, GZ, YJ, and DBSR: single nematode DNA from populations of *R. similis,* which originated from *Zingiber officinale* Roscoe (XIN), *Crataegus pinnatifida* (ML), *Musa* AAA Giant Cavendish cv.Baxi (HN6), *Maranta arundinacea* (HL), *Chrysalidocarpus lutescens* (SWK), *Citrus reticulata* (GJ), *Anthurium andraeanum* ‘Pink Champion’ (FZ), *Anthurium andraeanum* Linden (GZ), *Curcuma longa* (YJ), and *Anubias nana* (DBSR), respectively; M: DS2000 marker (GDSBio Co., Ltd., Guangzhou China); Fe: female; Ma: male; J2: second stage juvenile; Eg: egg

Single nematode DNA from 10 populations was used as the template, and sequence amplification of four *Rs-pel*s of the class III PEL family, i.e., *Rs-pel-2, Rs-pel-3, Rs-pel-4,* and *Rs-pel-5*, was performed. The results showed that *Rs-pel-2, Rs-pel-4,* and *Rs-pel-5* could be amplified from the SWK, GJ, FZ, GZ, DBSR, and YJ populations that parasitised rough lemon, and the XIN, ML, HN6, and HL populations that did not parasitise rough lemon. However, the gene *Rs-pel-3* could only be amplified from the six populations parasitizing rough lemon, showed stable expression in four developmental stages of these populations, and could not be amplified from the four non-parasitized rough lemon populations (Fig. 6A)*.* Therefore, *Rs-pel-3* could be used as a target gene to identify the banana and citrus pathotypes of *R. similis* and could be identified at all developmental stages in the citrus pathotype (Fig. 6B).

## Discussion

Two pathotypes, the banana and citrus pathotypes of *R. similis* are generally believed to exist with different host ranges and be morphologically indistinguishable. In this study, the parasitism and pathogenicity of 10 *R. similis* populations to rough lemon were determined using the method described by Kaplan et al. (1994) and the Pluronic F-127 gel system (Čepulytė et al. 2018; Wang et al. 2009). The results showed that six populations could parasitise rough lemon, whereas four populations could not. Four *Rs-pel*s of the Class III PEL family (*Rs-pel-2, Rs-pel-3, Rs-pel-4, Rs-pel-5*) could be amplified from six populations of parasitised rough lemon, whereas only three *Rs-pel* of the Class III PEL family (*Rs-pel-2, Rs-pel-4, Rs-pel-5*) could be amplified from four populations of non-parasitised rough lemon, and *Rs-pel-3* was stably expressed in four developmental stages of populations of parasitised rough lemon. *Rs-pel-3* was proven to be used as a target gene to distinguish the banana and citrus pathotypes of *R. similis*.

Kaplan and Opperman (1997) conducted parasitism and pathogenicity experiments on *R. similis* in citrus using the method described by Kaplan et al. (1994). They reported that if fewer than 10 nematodes were isolated from the rhizosphere (including in root and rhizosphere soil), the population would not parasitise citrus, whereas if more than 100 nematodes were isolated from the rhizosphere, the population would parasitise citrus (Kaplan and Opperman 1997). However, the parasitism of a population of 10–100 nematodes isolated from the rhizosphere has not been clearly defined by Kaplan and Opperman (1997). Goo and Sipes (1997) proposed that if low numbers of nematodes were recovered from the root, it suggested a low level of nematode reproduction might have occurred on the plant, indicating it was a poor host; if a few nematodes were recovered from the media but not from the root, it suggested that these nematodes might have been survivors from the inoculation, so the plant was considered a non-host or a very poor host. In this study, 30 days after rough lemons were inoculated with SWK, GJ, FZ, GZ, DBSR, and YJ populations of *R. similis* nematodes were isolated from each inoculated root system. The average number of nematodes in the roots was 8–20, and the total number of nematodes isolated from the rhizosphere of each inoculation treatment was more than 20. The roots of rough lemons inoculated with these six populations showed clear symptoms of damage, and the average nematode number in the root and rhizosphere soil was significantly higher than that of the control treatment and the other four populations. However, 30 days after rough lemon inoculation with XIN, ML, HN6, and HL populations, only 1–5 nematodes were isolated from seven inoculated root systems, and no nematodes were isolated from the other three inoculated root systems. The average nematode number in the roots was only one or two, and the total number of nematodes isolated from the rhizosphere was less than 10 in each inoculation treatment, both of which were not significantly different from those of the control. The roots of rough lemons inoculated with these four populations did not show symptoms of damage (Fig. 1).

In addition, the nematodes of SWK, GJ, FZ, GZ, DBSR, and YJ populations were observed to be attracted to and invaded the rough lemon roots, whereas the nematodes of XIN, ML, HN6, and HL populations were only attracted to but did not invade the rough lemon roots in the Pluronic F-127 gel system (Fig. 2). Plant root exudates can lead to tropism of plant-parasitic nematodes in both host and non-host plants (Sasakicrawley 2013; Hu et al. 2017); however, they can only induce nematodes to infect host plants and not non-host plants. Therefore, we considered that rough lemon was a host plant for the SWK, GJ, FZ, GZ, DBSR, and YJ populations, but not for the XIN, ML, HN6, and HL populations. This indicated that the SWK, GJ, FZ, GZ, DBSR, and YJ populations belonged to the citrus pathotype (or citrus race), whereas the XIN, ML, HN6, and HL populations belonged to the banana pathotype (or banana race).

Many studies have shown that *pel* is an important factor affecting host range. Different types of PELs recognise different sequences of methylated and unmethylated galacturonate sites with different chemical and enzyme properties (Herron et al. 2000; Filho et al. 2016), which may result in different pathogen host ranges. In this study, five *pel*s from *R. similis* (*Rs-pel-1, Rs-pel-2, Rs-pel--3, Rs-pel-4, Rs-pel-5*) were screened and cloned based on *R. similis* transcriptome data, four of which belonged to the Class III family (*Rs-pel-2, Rs-pel-3, Rs-pel-4, Rs-pel-5*). The amplification from genomic DNA and analysing gene expression of *pel*s in the 10 *R. similis* populations indicated that the gene *Rs-pel-3* could be amplified from six populations that parasitised rough lemon (SWK, GJ, FZ, GZ, DBSR, and YJ), but could not be amplified from four populations that did not parasitise rough lemon (XIN, ML, HN6, and HL). Therefore, the gene *Rs-pel-3* was absent in the genomes of non-citrus parasitic populations, and the expression of *Rs-pel-3* may be related to *R. similis* parasitism in *Citrus* spp.. Reportedly, the difference in *pel* expression in sedentary plant-parasitic nematodes is associated with their host range (Stare et al. 2011; Sabeh et al. 2019; Tian et al. 2019). Stare et al. (2011) reported that the difference in sequence polymorphism and gene copy of the *pel-2* gene from *Globodera* was associated with the variation in the host range of *G. rostochiensis*, *G. pallida*, and *G. tabacum*, while Sabeh et al. (2019) considered that the difference expression of *pel-1* was related to the host range of *G. rostochiensis*, *G. pallida, G. tabacum*, and *G. mexicana*. Tian et al. (2019) reported differences in *pel* expression between tobacco and soybean pathotypes of *Heterodera glycines*. This study revealed the relationship between the gene *Rs-pel-3* and host specialisation of *R. similis* and confirmed the difference between the two *R. similis* pathotypes at the molecular level. It is the first time that the difference in *pel* expression from migratory plant-parasitic nematodes may also be related to their host range.

Moreover, this study confirmed that the gene *Rs-pel-3* was stably expressed in eggs, juveniles, females, and males of six populations of *R. similis* parasitising rough lemon. Therefore, *Rs-pel-3* can not only be used as a molecular marker to distinguish citrus from banana pathotypes of *R. similis*, but also as a target gene to develop molecular identification methods for the quick and accurate identification of *R. similis* pathotypes.

## Supplementary information

ESM 1 (PDF 186 kb)
