# Supplementary material for: Pectate lyase genes from Radopholus similis and their application in pathotype identification
Source: Appl Microbiol Biotechnol. 2024 Apr 12;108(1):298. doi: 10.1007/s00253-024-13124-3 (PMC11009743; doi:10.1007/s00253-024-13124-3)
Supplement: Supplementary file 1 — (PDF 186 kb) [file 253_2024_13124_MOESM1_ESM.pdf]

Applied Microbiology and Biotechnology

Supplemental Material-Title page

Pectate lyase genes from *Radopholus similis* and their application in pathotype identification

Sihua Yang, Shuai Yang, Qianying Li, Yang Lu，Xin Huang, Chun Chen, Chunling Xu[#], Hui Xie[#]

Laboratory of Plant Nematology and Research Center of Nematodes of Plant Quarantine, Department of Plant Pathology, College of Plant Protection, South China Agricultural University, Guangzhou, 510642, People's Republic of China

#Correspondence authors: Hui Xie (E-mail: xiehui@scau.edu.cn) and Chunling Xu (E-mail: xuchunling@scau.edu.cn)

Supplemental Material-Table S1

Table S1 Primers used for PCR amplification of pectate lyase genes from *Radopholus similis*

Gene ID	Primer name	Primer sequence (5’-3’)	Use
Rs-pel-1	U1735GSP1	CGGATTCTCGTGGAAGTTGCGGATG	5'-RACE amplification
	U1735GSP2	AGACCTGCCACATCCCCTTCATCACG	3'-RACE amplification
	U1735NGSP1	GTTGCGGATGTTGACGGTGC	5'-RACE amplification
	Full-*Rs-pel1*-F	ATGCGGACGCTGGCGCGGC	PCR amplification
	Full-*Rs-pel1*-R	CTAATAAAATGGATTTGCTGCA	PCR amplification
Rs-pel-2	U3259GSP1	CGTTTCCCTCTCTACCGCCCCGACTA	5'-RACE amplification
	U3259GSP2	CCCACGCCAAAGAGCACTCAGGTCG	3'-RACE amplification
	U3259NGSP1	TGTTGCCCTCGTAGCCCTGA	5'-RACE amplification
	U3259NGSP2	AGGGTGGGTGCTGACTGTGG	3'-RACE amplification
	Full-*Rs-pel2*-F	ATGAGCCTGCACTTACTACTTTTGG	PCR amplification
	Full-*Rs-pel2*-R	CTAGTTGACGATTGTGATGTCGGC	PCR amplification
Rs-pel-3	U10880GSP1	CGCCGCCGATTTTCACGCTTTTCAGT	5'-RACE amplification
	U10880GSP2	GCGTCATTTTGGTGTGCGGCTCTTGC	3'-RACE amplification
	Full-*Rs-pel3*-F	ATGGAAAGTGCTACTTCAAGTCA	PCR amplification
	Full-*Rs-pel3*-R	TCATGAGAGTATGGTTACGTCCG	PCR amplification
Rs-pel-4	U11615GSP1	GCTGCCGCCCGAGTTGTTGCCGTC	5'-RACE amplification
	U11615GSP2	AACAACTCGGGCGGCAGCAAGGAT	3'-RACE amplification
	Full-*Rs-pel4*-F	ATGCAAAGCGCTTCTTCACTTG	PCR amplification
	Full-*Rs-pel4*-R	TTAGACCACTTTAACCGCGGAGG	PCR amplification
Rs-pel-5	U16802GSP1	GAAGTGCCTTTGCCGTTTTCGGTGGG	5'-RACE amplification
	U16802GSP2	TTCGCCCCGTCCACCTCCAGCAAG	3'-RACE amplification
	Full-*Rs-pel5*-F	ATGTACTCGCTCTTCGTCCTATC	PCR amplification
	Full-*Rs-pel5*-R	CTAGTTGACAATGGTGATAGAGGAG	PCR amplification
